# Real-world functional and anatomical outcomes of intravitreal aflibercept 8 mg in treatment-naïve neovascular age-related macular degeneration: a phenotype-based analysis by pachychoroid and polypoidal lesion status

**DOI:** 10.1186/s40942-026-00901-7

**Published:** 2026-07-15

**Authors:** Amiko Matsuyama, Akiko Miki, Toshiki Oka, Eisuke Ikeuchi, Maya Kishimoto-Kishi, Katsuaki Miki, Hisanori Imai, Makoto Nakamura

**Affiliations:** 1 Division of Ophthalmology, Department of Surgery, Kobe University Graduate School of Medicine, 7-5-2 Kusunoki-cho, Chuo-ku, Kobe, Hyogo 650-0017 Japan; 2 Department of Ophthalmology, Kansai Medical University Medical Center, 210-15, Fumizono-cho, Moriguchi-shi, Osaka 570-0074 Japan; 3 Department of Ophthalmology, Kansai Medical University, 2-3-1 Shinmachi, Hirakata-shi, Osaka 573-1191 Japan

**Keywords:** Aflibercept 8 mg, Neovascular age-related macular degeneration, Pachychoroid neovasculopathy, Polypoidal choroidal vasculopathy, Real-world study, Subretinal hyperreflective material

## Abstract

**Background:**

To evaluate the short-term functional and anatomical outcomes of intravitreal aflibercept 8 mg in treatment-naïve neovascular age-related macular degeneration (nAMD), and to explore whether the early treatment response varied according to pachychoroid and polypoidal lesion status.

**Methods:**

This retrospective, multicenter observational study evaluated 45 treatment-naïve eyes of 45 patients with nAMD who received three monthly loading injections of aflibercept 8 mg. Best-corrected visual acuity (BCVA), central retinal thickness (CRT), central choroidal thickness (CCT), and exudative findings (subretinal fluid [SRF], intraretinal fluid [IRF], and subretinal hyperreflective material [SHRM]) were assessed at baseline and at 1, 2, and 4 months. Pachychoroid and polypoidal lesion status was determined using multimodal imaging. Longitudinal changes were analyzed using linear mixed-effects models, with subgroup analyses by pachychoroid and polypoidal lesion status.

**Results:**

At 4 months, mean BCVA improved from 0.27 ± 0.33 to 0.20 ± 0.31 logMAR (*P* = 0.005), CRT decreased from 382.1 ± 189.7 to 231.3 ± 109.1 μm, and CCT from 263.0 ± 146.5 to 231.0 ± 127.2 μm (both *P* < 0.001). The dry macula rate (absence of both SRF and IRF) increased from 17.8% to 75.6% (*P* < 0.001). Functional and retinal anatomical responses were broadly consistent across phenotypes, with no significant time-by-phenotype interactions for BCVA or CRT. Pachychoroid eyes showed a greater CCT reduction (interaction *P* < 0.001), but this was no longer significant after adjusting for baseline CCT. As exploratory observations, the time-by-polypoidal-lesion interaction for BCVA was not significant (*P* = 0.052), and residual SHRM at 4 months was present in 0% of polypoidal-lesion eyes versus 22.2% of those without (*P* = 0.067).

**Conclusions:**

Intravitreal aflibercept 8 mg led to significant short-term improvements in BCVA, CRT, CCT, and exudative findings in treatment-naïve nAMD, with benefit observed irrespective of pachychoroid or polypoidal lesion status.

## Background

Neovascular age-related macular degeneration (nAMD) is one of the main causes of irreversible vision loss among older adults globally [1, 2]. Intravitreal anti-vascular endothelial growth factor (VEGF) therapy is the established first-line therapy for nAMD, demonstrating significant improvements in visual and anatomical outcomes in randomized trials [3, 4]. However, the need for frequent intravitreal injections remains a considerable burden for patients, caregivers, and healthcare systems and limits sustained disease management in real-world practice [5].

Aflibercept 8 mg is a high-dose formulation (114.3 mg/mL, 0.07 mL) developed to achieve more prolonged intraocular VEGF suppression through a four-fold increase in the administered dose while preserving the molecular structure of the original 2 mg formulation. In the phase 3 PULSAR trial, aflibercept 8 mg administered every 12 or 16 weeks following three initial monthly loading doses demonstrated non-inferior best-corrected visual acuity (BCVA) gains compared with aflibercept 2 mg every 8 weeks at week 48 (mean change + 6.7 and + 6.2 letters vs. +7.6 letters, respectively) [6], with these gains maintained through week 96 [7]. In the earlier phase 2 cd trial, the proportion of fluid-free eyes at week 16 was slightly higher with aflibercept 8 mg than with aflibercept 2 mg, although the difference was not statistically significant [8]. These findings suggest that a higher dose of aflibercept may achieve sustained disease control while enabling extended dosing intervals. Nevertheless, real-world evidence regarding the short-term efficacy of aflibercept 8 mg in treatment-naïve patients remains limited, with most reports from single-center cohorts [9–12].

Notably, nAMD is clinically heterogeneous and includes subtypes such as pachychoroid neovasculopathy (PNV) and polypoidal choroidal vasculopathy (PCV), which differ substantially in choroidal thickness, lesion morphology, and treatment response [13–15]. These subtypes are especially common in Asian populations, where they represent a large proportion of nAMD cases [16–18]. The therapeutic effect of aflibercept 8 mg across these subtypes remains unclear. Several subgroup analyses of the PULSAR trial have demonstrated efficacy consistent with the overall trial results in subgroups defined by baseline clinical characteristics [19], Japanese ethnicity [20], and broader Asian ethnicity [21]. However, none of these analyses examined outcomes by pachychoroid or polypoidal lesion status.

Therefore, we conducted this real-world, multicenter study to evaluate the short-term functional and anatomical outcomes of intravitreal aflibercept 8 mg in treatment-naïve nAMD, and to explore whether the early therapeutic response differs by pachychoroid or polypoidal lesion status.

## Methods

This retrospective, observational, multicenter study was conducted at two hospitals (Kobe University Hospital and Kansai Medical University Medical Center) in Japan. The study protocol was approved by the Institutional Review Board of Kobe University Hospital (approval number: B250205) and conducted in accordance with the tenets of the Declaration of Helsinki. Given the retrospective design, the requirement for written informed consent was waived in favor of an opt-out procedure via public disclosure on the institution’s website. This study was reported in accordance with the Strengthening the Reporting of Observational Studies in Epidemiology (STROBE) statement.

Consecutive patients with treatment-naïve nAMD who initiated intravitreal aflibercept 8 mg therapy between June 2024 and June 2025 at participating institutions were identified from electronic medical records. During the study period, all consecutive treatment-naïve eyes that initiated aflibercept 8 mg therapy met the eligibility criteria; a total of 45 eyes from 45 patients were included in the final analysis. The inclusion criteria were as follows: (1) diagnosis of treatment-naïve nAMD (including PNV and PCV) based on multimodal imaging, such as spectral-domain optical coherence tomography (SD-OCT), fluorescein angiography (FA), and indocyanine green angiography (ICGA); (2) completion of three consecutive monthly loading injections of aflibercept 8 mg; and (3) follow-up of at least four months after the initial injection. The exclusion criteria were as follows: (1) any prior anti-VEGF therapy, photodynamic therapy, laser photocoagulation, or vitrectomy in the study eye; (2) choroidal neovascularization due to causes other than AMD (e.g., pathological myopia, angioid streaks, or uveitis); (3) significant media opacity precluding OCT evaluation.

All patients received three monthly intravitreal injections of aflibercept 8 mg (114.3 mg/mL, 0.07 mL; Bayer AG, Leverkusen, Germany) as a loading phase. Injections were performed under sterile conditions using topical anesthesia and povidone-iodine preparation.

Comprehensive ophthalmologic examinations were performed at baseline and at 1, 2, and 4 months following the initiation of treatment. The 4-month visit corresponded to the first post-loading follow-up visit. BCVA was measured using a Landolt-C chart and converted to the logarithm of the minimum angle of resolution (logMAR) for statistical analysis. SD-OCT imaging was obtained using the Heidelberg Spectralis HRA + OCT (Heidelberg Engineering, Heidelberg, Germany) in enhanced-depth imaging (EDI) mode for choroidal visualization. CRT was defined as the distance manually measured from the internal limiting membrane to the retinal pigment epithelium at the fovea. CCT was manually measured from the outer border of the retinal pigment epithelium to the choroidoscleral junction at the fovea. Both CRT and CCT were measured on horizontal and vertical B-scans through the fovea by a single retinal specialist masked to clinical information, and the mean of the horizontal and vertical measurements was used for analysis. The presence of intraretinal fluid (IRF), subretinal fluid (SRF), and subretinal hyperreflective material (SHRM) was qualitatively assessed using SD-OCT at each visit.

PNV was diagnosed based on multimodal imaging findings, including dilated outer choroidal vessels (pachyvessels) on EDI-OCT, attenuation of the choriocapillaris overlying pachyvessels, and/or choroidal vascular hyperpermeability on mid-phase ICGA, in accordance with the Japanese clinical guidelines for neovascular age-related macular degeneration [22] and our previous reports [23, 24]. PCV was diagnosed based on the presence of nodular hyperfluorescent polypoidal lesions on ICGA with an associated branching vascular network, in accordance with the same guidelines [22]. Type 1, 2, and 3 macular neovascularization (MNV) were also classified according to the same guidelines [22]. All imaging-based classifications and diagnoses, as well as the qualitative assessment of SRF, IRF, and SHRM, were performed independently by two retinal specialists, with disagreements resolved by adjudication by a third retinal specialist.

The primary outcome was the longitudinal change in BCVA (logMAR) over 4 months. The secondary outcomes included longitudinal changes in CRT and CCT and the proportions of eyes with SRF, IRF, dry macula (absence of both SRF and IRF), and SHRM at each visit. Subgroup analyses compared baseline characteristics, longitudinal changes in BCVA, CRT, and CCT, as well as the dry macula rate and the proportion of eyes with residual SHRM at 4 months, between eyes with and without pachychoroid features and between eyes with and without polypoidal lesions. Univariable analyses were performed to identify baseline factors associated with achievement of dry macula at 4 months. Subgroup analyses by pachychoroid and polypoidal lesion status were exploratory and hypothesis-generating. Safety was assessed throughout follow-up by reviewing medical records for non-infectious intraocular inflammation, retinal vasculitis, and other serious ocular adverse events.

Baseline characteristics were compared between subgroups (pachychoroid status, polypoidal lesion status) using the Mann–Whitney U test for continuous variables (since several variables showed non-normal distribution on the Shapiro–Wilk test) and Fisher’s exact test for categorical variables. Longitudinal changes in BCVA, CRT, and CCT were analyzed using linear mixed-effects models (restricted maximum likelihood, AR(1) covariance structure selected based on the lowest AIC), with fixed effects of time (categorical: baseline, 1, 2, and 4 months), pachychoroid status, polypoidal lesion status, and the interaction terms between time and phenotype. Pairwise comparisons of estimated marginal means (EMM) between time points and between subgroups were performed using Bonferroni correction. Sensitivity analyses were performed by (1) adding the participating hospital as an additional fixed effect, (2) analyzing each phenotype separately, and (3) adding age and sex as covariates. Proportions of eyes with SRF, IRF, dry macula, and SHRM between baseline and 4 months were compared using McNemar’s test (exact); residual SHRM at 4 months was compared between polypoidal lesion subgroups using Fisher’s exact test. Baseline factors associated with achievement of dry macula at 4 months were explored using the Mann–Whitney U test (continuous) and Fisher’s exact test (categorical). As an additional analysis, post-treatment CCT at months 1, 2, and 4 was analyzed using a linear mixed-effects model with baseline CCT as a covariate; time, pachychoroid status, and their interaction as fixed effects; and each eye as a random effect. For the exploratory analysis of dry macula at 4 months, odds ratios with 95% confidence intervals were calculated for categorical baseline factors; for one factor with a zero cell, the odds ratio was not estimable. All analyses were performed using SPSS version 29.0 (IBM, Armonk, NY, USA), and a P value < 0.05 was considered statistically significant. There were no missing data; therefore, no imputation was performed.

## Results

A total of 45 eyes from 45 patients were included in the analysis. The mean age was 76.6 ± 11.5 years, and 32 patients were male. Pachychoroid features were observed in 20 eyes (44.4%), and polypoidal lesions were identified in 18 eyes (40.0%); 5 eyes (11.1%) had both pachychoroid features and polypoidal lesions. At baseline, the mean visual acuity was 0.27 ± 0.33 logMAR, mean CRT was 382.1 ± 189.7 μm, and mean CCT was 263.0 ± 146.5 μm (Table 1). Data were complete for all 45 eyes at all four time points, with no missing data.

**Table 1:** Baseline patient characteristics

Characteristic	Value
Number of eyes	45 (45 patients)
Sex (male/female), n	32/13
Age (years)	76.6 ± 11.5
Subtype (type1/2/1 + 2/3/PCV), n	16/7/3/1/18
Pachychoroid (+/–), n	20/25
Polypoidal lesions (+/–), n	18/27
BCVA (logMAR)	0.27 ± 0.33
Central retinal thickness (µm)	382.1 ± 189.7
Central choroidal thickness (µm)	263.0 ± 146.5

Values are presented as mean ± standard deviation. PCV: polypoidal choroidal vasculopathy, BCVA: best-corrected visual acuity, logMAR: logarithm of the minimum angle of resolution

Eyes with pachychoroid features were significantly younger (71.7 ± 13.9 vs. 80.5 ± 7.7 years, *P* = 0.020) and had a thicker choroid (345.3 ± 163.1 vs. 197.2 ± 94.9 μm, *P* < 0.001) than those without, whereas other baseline characteristics did not differ significantly between subgroups (Table 2). Eyes with polypoidal lesions had a higher proportion of male patients (male: 16/18 [88.9%] vs. 16/27 [59.3%], *P* = 0.046), without significant differences in other baseline characteristics. The proportions of eyes with SRF, IRF, and SHRM at baseline did not differ significantly according to pachychoroid or polypoidal lesion status (Table 2).

**Table 2:** Baseline clinical characteristics according to pachychoroid phenotype and presence of polypoidal lesions

	Pachychoroid (+)	Pachychoroid (–)	*P*-value	Polypoidal lesions (+)	Polypoidal lesions (–)	*P*-value
Sex (male/female), *n*	16/4	16/9	0.327	16/2	16/11	0.046*
Age (years)	71.7 ± 13.9	80.5 ± 7.7	0.020*	77.4 ± 8.6	76.1 ± 13.4	0.918
BCVA (logMAR)	0.37 ± 0.44	0.18 ± 0.21	0.189	0.22 ± 0.23	0.30 ± 0.40	0.918
Central retinal thickness (µm)	424.6 ± 240.9	348.2 ± 137.3	0.213	337.1 ± 85.3	412.1 ± 234.9	0.568
Central choroidal thickness (µm)	345.3 ± 163.1	197.2 ± 94.9	< 0.001*	238.7 ± 130.7	279.2 ± 159.0	0.354
SRF (+/–), n (%)	14/6 (70.0)	14/11 (56.0)	0.372	13/5 (72.2)	15/12 (55.6)	0.351
IRF (+/–), n (%)	6/14 (30.0)	8/17 (32.0)	1.000	5/13 (27.8)	9/18 (33.3)	0.753
SRF and/or IRF (+/–), n (%)	17/3 (85.0)	20/5 (80.0)	0.716	17/1 (94.4)	20/7 (74.1)	0.119
SHRM (+/–), n (%)	10/10 (50.0)	7/18 (28.0)	0.216	6/12 (33.3)	11/16 (40.7)	0.757

Values are presented as mean ± standard deviation or n (%). BCVA: best-corrected visual acuity, logMAR: logarithm of the minimum angle of resolution, SRF: subretinal fluid, IRF: intraretinal fluid, SHRM: subretinal hyperreflective material. Categorical variables were compared using Fisher’s exact test, and continuous variables were compared using the Mann–Whitney U test. *P < 0.05

**Fig. 1:**
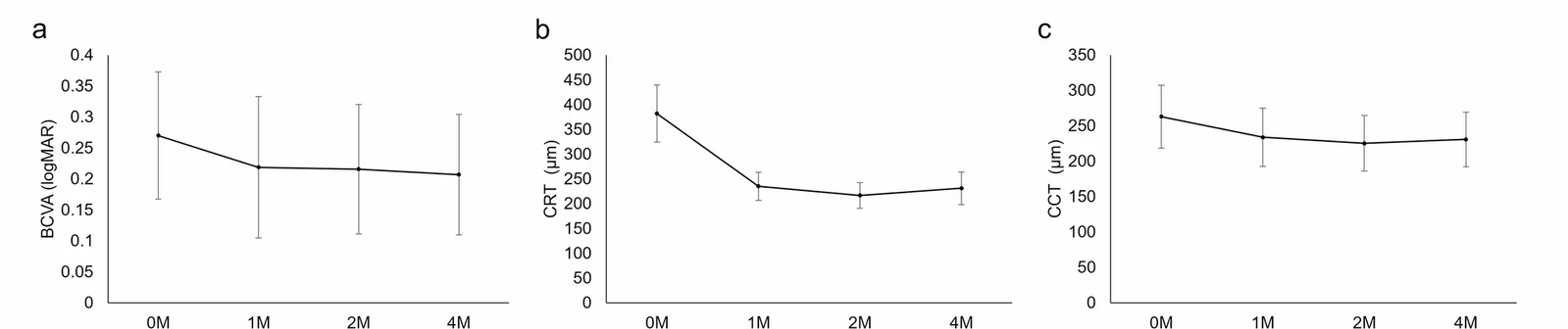
Longitudinal changes in BCVA, CRT, and CCT. (**a**) Mean best-corrected visual acuity in logMAR, (**b**) mean central retinal thickness, and (**c**) mean central choroidal thickness at baseline, 1 month, 2 months, and 4 months. Linear mixed-effects model analysis demonstrated a significant effect of time on all three parameters (BCVA, *P* = 0.009; CRT, *P* < 0.001; CCT, *P* < 0.001). Data are presented as means with 95% confidence intervals (error bars). BCVA, best-corrected visual acuity; CCT, central choroidal thickness; CRT, central retinal thickness; logMAR, logarithm of the minimum angle of resolution

Figure 1 shows the longitudinal changes in BCVA, CRT, and CCT. The mean BCVA improved from 0.27 ± 0.33 logMAR at baseline to 0.22 ± 0.37 at 1 month, 0.21 ± 0.34 at 2 months, and 0.20 ± 0.31 at 4 months. CRT decreased from 382.1 ± 189.7 μm at baseline to 235.3 ± 92.5 μm at 1 month, 217.1 ± 85.2 μm at 2 months, and 231.3 ± 109.1 μm at 4 months. CCT decreased from 263.0 ± 146.5 μm at baseline to 233.9 ± 135.0 μm at 1 month, 225.5 ± 129.2 μm at 2 months, and 231.0 ± 127.2 μm at 4 months. Mixed-effects model analysis demonstrated a significant effect of time on BCVA (*P* = 0.009), CRT (*P* < 0.001), and CCT (*P* < 0.001). In Bonferroni-adjusted pairwise comparisons of estimated marginal means, BCVA improved significantly from baseline only at 4 months (mean difference − 0.080; 95% CI, -0.128 to -0.031, *P* = 0.005), whereas CRT and CCT decreased significantly from baseline at all post-treatment time points (CRT: -149.8, -167.7, and − 157.1 μm (95% CI: -188.7 to -110.8, -206.7 to -128.8, and − 196.0 to -118.1, respectively) at 1, 2, and 4 months, respectively, all *P* < 0.001; CCT: -30.3, -39.3, and − 34.3 μm (95% CI: -39.5 to -21.0, -48.5 to -30.1, and − 43.5 to -25.1, respectively), respectively, all *P* < 0.001).

**Fig. 2:**
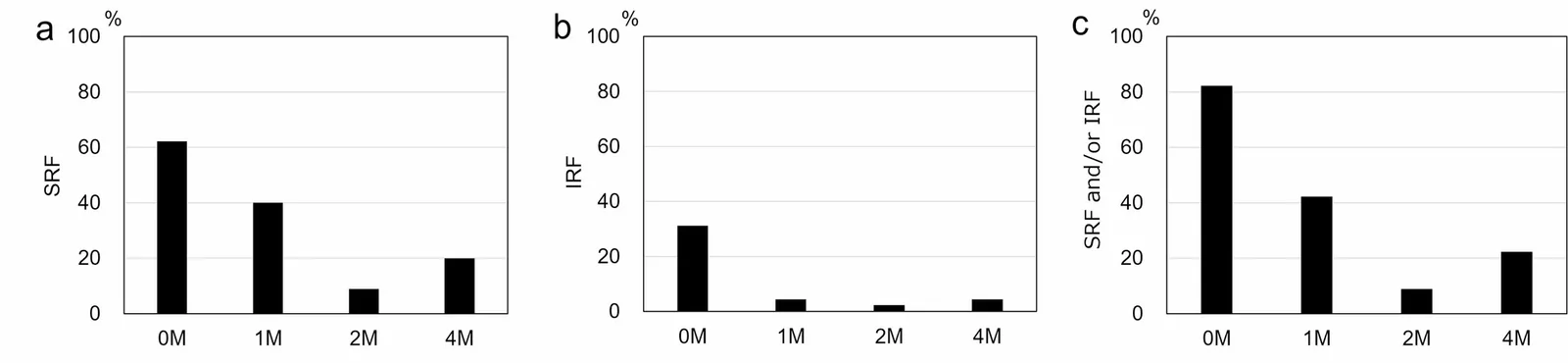
Longitudinal changes in retinal fluid status. The proportions of eyes with (**a**) subretinal fluid, (**b**) intraretinal fluid, and (**c**) eyes with either subretinal fluid or intraretinal fluid (a dry macula was defined as the absence of both) at baseline, 1 month, 2 months, and 4 months are shown. The proportions at 4 months were significantly lower than those at baseline for all three categories (*P* < 0.001 for each, McNemar’s exact test). IRF: intraretinal fluid; SRF: subretinal fluid

The longitudinal changes in retinal fluid status are shown in Fig. 2. The proportion of eyes with SRF decreased from 62.2% (28/45) at baseline to 22.2% (10/45) at 4 months. The proportion of eyes with IRF decreased from 31.1% (14/45) to 4.4% (2/45) over the same period. The dry macula rate (absence of both SRF and IRF) increased from 17.8% (8/45) at baseline to 86.7% (39/45) at 2 months and 75.6% (34/45) at 4 months. Each of these changes between baseline and 4 months was statistically significant (*P* < 0.001 for each, McNemar’s exact test); however, the difference in dry macula rate between 2 and 4 months was not significant (*P* = 0.180). Between months 2 and 4, 7 of the 39 eyes with a dry macula at month 2 developed recurrent SRF and/or IRF, whereas 2 of the 6 eyes without a dry macula achieved a dry macula, so that the number of eyes with a dry macula decreased from 39 to 34. The proportion of eyes with SHRM decreased from 37.8% (17/45) at baseline to 13.3% (6/45) at 4 months (*P* < 0.001, McNemar’s exact test).

**Fig. 3:**
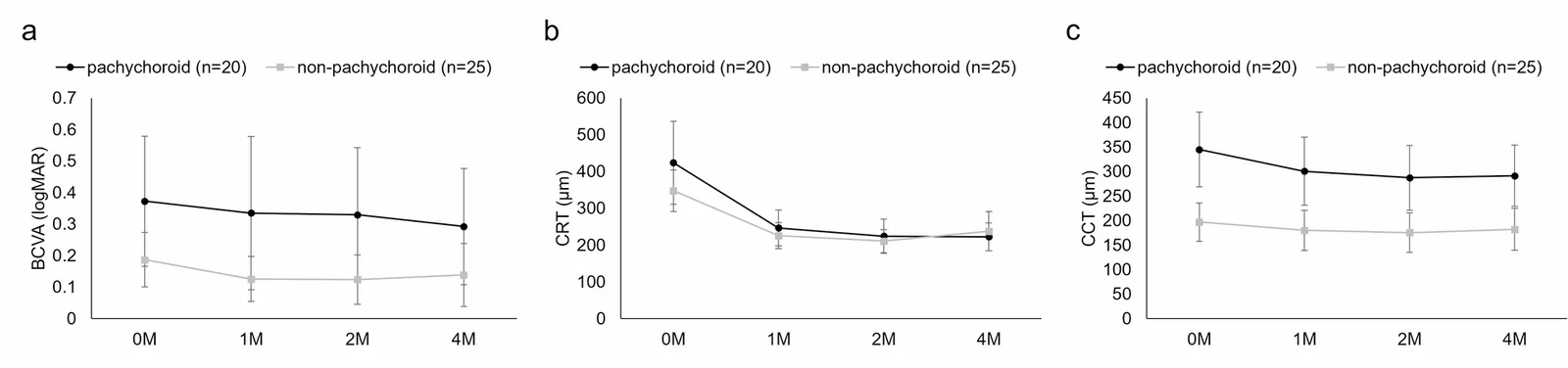
Longitudinal changes in BCVA, CRT, and CCT according to pachychoroid status. (**a**) Mean best-corrected visual acuity in logMAR, (**b**) mean central retinal thickness, and (**c**) mean central choroidal thickness in eyes with pachychoroid features (*n* = 20) and without pachychoroid features (*n* = 25) at baseline, 1, 2, and 4 months. Linear mixed-effects model analysis showed no significant interaction between time and pachychoroid status for BCVA (*P* = 0.315) or CRT (*P* = 0.110). In contrast, the interaction between time and pachychoroid status for CCT was significant (*P* < 0.001). Data are presented as means with 95% confidence intervals (error bars). BCVA, best-corrected visual acuity; CCT, central choroidal thickness; CRT, central retinal thickness; logMAR, logarithm of the minimum angle of resolution

Figure 3 presents the longitudinal changes in BCVA, CRT, and CCT by pachychoroid status. Both the pachychoroid and non-pachychoroid groups demonstrated improvements in BCVA and reductions in CRT over time. Mixed-effects model analysis showed no significant interaction between time and pachychoroid status for BCVA (*P* = 0.315) or CRT (*P* = 0.110). In Bonferroni-adjusted within-group comparisons, BCVA improved significantly from baseline to 4 months in the pachychoroid group (mean difference − 0.113; 95% CI, -0.188 to -0.039, *P* = 0.012) but not in the non-pachychoroid group (mean difference − 0.046; 95% CI, -0.109 to + 0.017; *P* = 0.778), whereas CRT decreased significantly at all post-treatment time points in both groups (all *P* < 0.001).

For CCT, a significant interaction between time and pachychoroid status (*P* < 0.001) and a significant main effect of pachychoroid status (*P* = 0.003) were observed. Pachychoroid eyes had a thicker choroid than non-pachychoroid eyes at all time points (between-group *P* < 0.001 at baseline, *P* = 0.004 at 1 month, *P* = 0.007 at 2 months, and *P* = 0.009 at 4 months). Within-group analysis showed that CCT decreased significantly from baseline at all post-treatment time points in pachychoroid eyes (mean differences − 43.5, -57.1, and − 53.9 μm (95% CI: -57.7 to -29.3, -71.3 to -42.9, and − 68.1 to -39.7, respectively) at 1, 2, and 4 months, respectively; all *P* ≤ 0.001), whereas smaller reductions were observed in non-pachychoroid eyes, reaching statistical significance only at 2 months (-21.5 μm [95% CI, -33.5 to -9.6], *P* = 0.039) and borderline at 4 months (-14.7 μm [95% CI, -26.7 to -2.8], *P* = 0.053). After adjustment for baseline CCT, the time-by-pachychoroid interaction for CCT was no longer significant (*P* = 0.18), indicating that the greater CCT reduction in pachychoroid eyes was largely attributable to their thicker baseline choroid.

**Fig. 4:**
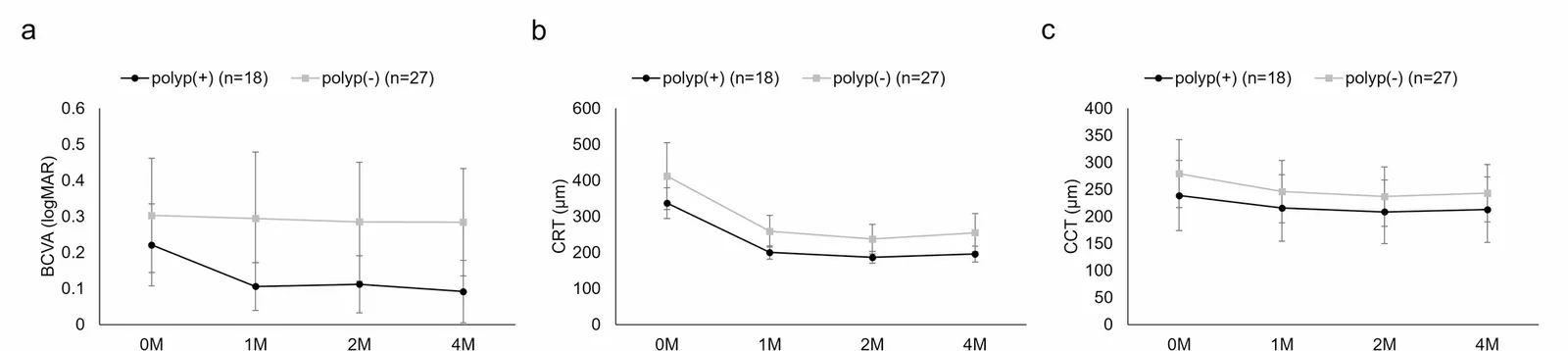
Longitudinal changes in BCVA, CRT, and CCT according to polypoidal lesion status. (**a**) Mean best-corrected visual acuity in logMAR, (**b**) mean central retinal thickness, and (**c**) mean central choroidal thickness in eyes with polypoidal lesions (*n* = 18) and without polypoidal lesions (*n* = 27) at baseline, 1 month, 2 months, and 4 months after treatment. Linear mixed-effects model analysis showed no significant interaction between time and polypoidal lesion status for CRT (*P* = 0.971) or CCT (*P* = 0.985). The interaction between time and polypoidal lesion status for BCVA was borderline (*P* = 0.052). Data are presented as means with 95% confidence intervals (error bars). BCVA, best-corrected visual acuity; CCT, central choroidal thickness; CRT, central retinal thickness; logMAR, logarithm of the minimum angle of resolution

The longitudinal changes in BCVA, CRT, and CCT by polypoidal lesion status are demonstrated in Fig. 4. Both groups showed improvements in BCVA and reductions in CRT and CCT over time. No significant interaction between time and polypoidal lesion status was observed for CRT (*P* = 0.971) or CCT (*P* = 0.985). The interaction between time and polypoidal lesion status for BCVA was borderline and did not reach significance (*P* = 0.052). In Bonferroni-adjusted within-group comparisons, BCVA improved significantly from baseline to 4 months in eyes with polypoidal lesions (mean difference − 0.144; 95% CI, -0.222 to -0.067, *P* < 0.001), but not in those without (mean difference − 0.015; 95% CI, -0.076 to + 0.046; *P* = 1.000). CRT and CCT decreased significantly from baseline at all post-treatment time points in both groups (all *P* ≤ 0.004).

Although the proportions of eyes with SRF, IRF, and SHRM at baseline did not differ significantly between the polypoidal lesion subgroups (Table 2), at 4 months SHRM was absent in all 18 eyes with polypoidal lesions (0%) but present in 6 of 27 eyes without polypoidal lesions (22.2%; *P* = 0.067, Fisher exact test; Table 3). In contrast, the proportions of eyes with residual SRF and IRF at 4 months did not differ significantly between pachychoroid subgroups (*P* = 0.147 and 1.000, respectively) or between polypoidal lesion subgroups (*P* = 0.716 and 0.509, respectively; data not shown).

**Table 3:** Treatment outcomes at 4 months according to pachychoroid phenotype and presence of polypoidal lesions

	Pachychoroid (+)	Pachychoroid (–)	*P*-value	Polypoidal lesions (+)	Polypoidal lesions (–)	*P*-value
BCVA (logMAR)	0.29 ± 0.39	0.14 ± 0.24	0.118	0.09 ± 0.17	0.28 ± 0.38	0.090
Dry macula rate (%)	85.0	68.0	0.297	83.3	70.4	0.482
Central retinal thickness (µm)	222.8 ± 81.1	238.2 ± 130.3	0.736	195.9 ± 43.8	254.9 ± 133.7	0.308
Central choroidal thickness (µm)	291.7 ± 134.2	182.5 ± 102.8	0.001*	212.6 ± 121.5	243.3 ± 134.1	0.410
SHRM (+/–), n (%)	3/17 (15.0)	3/22 (12.0)	1.000	0/18 (0.0)	6/21 (22.2)	0.067

Values are presented as mean ± standard deviation or n (%). BCVA: best-corrected visual acuity, logMAR: logarithm of the minimum angle of resolution, CRT: central retinal thickness, CCT: central choroidal thickness, SHRM: subretinal hyperreflective material. Categorical variables were compared using Fisher’s exact test, and continuous variables were compared using the Mann–Whitney U test. Model-based estimated marginal mean changes from baseline and the formal tests for differential longitudinal treatment response (time-by-phenotype interaction terms and, for CCT, the baseline-CCT-adjusted analysis) are reported in the Results. *P < 0.05

The time effects and interactions for both pachychoroid and polypoidal lesion status remained unchanged after adjustment for the participating hospital (BCVA: time, *P* = 0.009; interaction with pachychoroid status, *P* = 0.314; interaction with polypoidal lesion status, *P* = 0.052; CRT: time, *P* < 0.001; interaction with pachychoroid status, *P* = 0.112; interaction with polypoidal lesion status, *P* = 0.972; CCT: time, *P* < 0.001; interaction with pachychoroid status, *P* < 0.001; interaction with polypoidal lesion status, *P* = 0.985). These results indicate that the treatment responses and subgroup differences were not attributable to inter-hospital variability. The results also remained consistent when each phenotype was analyzed separately. The interaction effects remained consistent after adjustment for age and sex as covariates.

An exploratory analysis was performed to identify baseline factors associated with achievement of a dry macula at 4 months. Of the 45 eyes included, 34 (75.6%) achieved a dry macula at 4 months, and 11 (24.4%) did not. In this exploratory analysis, none of the baseline factors was significantly associated with achievement of a dry macula at 4 months (all *P* > 0.05; Table 4).

**Table 4:** Association between baseline clinical factors and achievement of dry macula at 4 months

	Dry macula (+)	Dry macula (–)	*P*-value	OR (95% CI)
Sex (male/female)	25/9	7/4	0.704	1.59 (0.37–6.74)
Age (years)	78.2 ± 11.2	71.5 ± 11.9	0.081	—
Pachychoroid (+/–)	17/17	3/8	0.297	2.67 (0.60–11.80)
Polypoidal lesions (+/–)	15/19	3/8	0.482	2.11 (0.47–9.34)
BCVA (logMAR)	0.31 ± 0.38	0.15 ± 0.17	0.242	—
Central retinal thickness (µm)	393.8 ± 214.2	346.1 ± 93.2	0.937	—
Central choroidal thickness (µm)	262.5 ± 141.1	264.7 ± 175.5	0.833	—
SRF (+/–)	19/15	9/2	0.165	0.28 (0.05–1.50)
IRF (+/–)	10/24	4/7	0.717	0.73 (0.17–3.06)
SRF and/or IRF (+/–)	26/8	11/0	0.169	—
SHRM (+/–)	12/22	5/6	0.722	0.65 (0.16–2.60)

BCVA: best-corrected visual acuity, logMAR: logarithm of the minimum angle of resolution, SRF: subretinal fluid, IRF: intraretinal fluid, SHRM: subretinal hyperreflective material. Values are presented as mean ± standard deviation. Categorical variables were compared using Fisher’s exact test, and continuous variables were compared using the Mann–Whitney U test. Odds ratios (OR) with 95% confidence intervals are shown for categorical factors; the odds ratio for the presence of SRF and/or IRF at baseline was not estimable because of a zero cell (—). Continuous factors are not applicable (—)

None of the 45 eyes developed non-infectious intraocular inflammation, retinal vasculitis, or other serious ocular adverse events during the 4-month follow-up.

## Discussion

In this multicenter, retrospective study of 45 treatment-naïve eyes with nAMD, three monthly loading injections of intravitreal aflibercept 8 mg led to significant improvements in BCVA, substantial reductions in retinal and choroidal thickness, and pronounced suppression of exudative activity at 4 months. Retinal anatomical changes were similar between subgroups based on pachychoroid and polypoidal lesion status, whereas the choroidal thickness response differed significantly according to pachychoroid status, although this difference was largely attributable to the greater baseline choroidal thickness in pachychoroid eyes. The time-by-polypoidal-lesion interaction for BCVA was borderline and did not reach significance (*P* = 0.052), although within-group analyses showed a greater BCVA improvement in eyes with polypoidal lesions, in which residual SHRM was absent at 4 months. Taken together, aflibercept 8 mg produced significant functional and anatomical improvement that was broadly consistent across pachychoroid and polypoidal phenotypes; the phenotype-related observations were exploratory and hypothesis-generating. To our knowledge, this is the first real-world study to evaluate aflibercept 8 mg outcomes by pachychoroid and polypoidal lesion status in an Asian treatment-naïve nAMD cohort, using longitudinal mixed-effects models to characterize treatment responses over time.

Our findings are consistent with recent real-world reports of aflibercept 8 mg in treatment-naïve nAMD from Japan and Europe. Japanese studies by Hosoda et al. [10] (21 eyes) and Matsumoto et al. [9] (70 eyes), both evaluating eyes that completed three loading injections, reported significant BCVA improvement and reductions in central retinal thickness and choroidal thickness. The dry macula rate increased over time in both reports: 62%, 95%, and 100% at 1, 2, and 3 months in Hosoda et al., and 50.0%, 71.4%, and 82.9% at weeks 4, 8, and 12 in Matsumoto et al. European studies by Cattaneo et al. [11], Bartolomeo et al. [12], and Veritti et al. [25] showed significant reductions in central subfield thickness, BCVA gains, and rapid IRF and SRF volume reductions using AI-assisted OCT quantification. In our cohort, the significant reductions in retinal and choroidal thickness, the substantial reduction in exudative findings, and a dry macula rate of 86.7% at 2 months demonstrated early efficacy comparable to these reports. The dry macula rate at 2 months in our cohort was slightly lower than the 95% reported by Hosoda et al. but higher than the 71.4% reported by Matsumoto et al. at the same time point. However, although we did not evaluate at week 12, the dry macula rate in our cohort had declined to 75.6% at 4 months, lower than the 100% reported by Hosoda et al. at 3 months and the 82.9% reported by Matsumoto et al. at week 12. In our cohort, the proportion of eyes with SRF was lowest at 2 months and slightly increased by 4 months. The decrease in dry macula rate from 2 to 4 months was not statistically significant (McNemar’s exact test, *P* = 0.180); this decline likely reflects early recurrence of subretinal and/or intraretinal fluid in some eyes after completion of the three monthly loading injections, when the interval before the next injection became longer. However, our 4-month dry macula rate is not directly comparable with the 3-month rates in these reports, because our assessment was performed one month later, after the loading phase, when recurrent fluid can lower the dry macula rate.

In the ALTAIR study, a prospective Japanese trial of intravitreal aflibercept 2 mg using a treat-and-extend regimen, 35.7% of treatment-naïve eyes had IRF and 85.2% had SRF at baseline, which decreased to 11.8% and 31.7%, respectively, at week 16 after the loading phase, with 58.1% of eyes showing no retinal fluid at that time point [26]. Although this does not constitute a direct comparison, at the same time point, our cohort showed IRF and SRF decreasing from baseline to 4 months from 31.1% to 4.4% and from 62.2% to 22.2%, respectively, with the dry macula rate increasing from 17.8% to 75.6%; however, these differences should be interpreted only as indirect, cross-study observations. Furthermore, Rinaldi et al. [27] compared aflibercept 8 mg and 2 mg in a prospective pilot study (*n* = 15 each) and reported significantly greater reductions in central macular thickness (*P* = 0.008) and BCVA improvement (*P* = 0.012) with the 8 mg dose at 4 months. Although these pilot comparative data are compatible with robust early drying after aflibercept 8 mg, our study did not include an aflibercept 2 mg comparator arm; therefore, any inference regarding dose-related superiority should be regarded as indirect and requires confirmation in adequately powered head-to-head studies. An ALTAIR sub-analysis further showed that the presence or absence of retinal fluid at week 16 during aflibercept 2 mg therapy predicts both BCVA outcomes and the last treatment interval through 2 years [26]. These findings may be relevant to future studies evaluating individualized dosing-interval decisions after the loading phase.

Reductions in retinal thickness were observed in both groups, without significant differences in the time course by pachychoroid or polypoidal lesion status. In contrast, the choroidal thickness response differed substantially according to pachychoroid status. Pachychoroid eyes had a thicker choroid at baseline and showed greater reductions in choroidal thickness during follow-up compared with non-pachychoroid eyes (interaction between time and pachychoroid status, *P* < 0.001). In pachychoroid-spectrum disease, structural and functional choroidal abnormalities such as choroidal vascular hyperpermeability and pachyvessel dilatation are considered the major pathophysiological contributors [13, 18, 22]. Although CCT decreased significantly in pachychoroid eyes, the greater reduction was attenuated after adjustment for baseline CCT, indicating that this finding should be interpreted primarily in the context of greater baseline CCT rather than as definitive evidence of a phenotype-specific choroidal effect. However, the smaller choroidal thickness reduction in non-pachychoroid eyes may also be partly explained by their thinner baseline choroid, which provides limited capacity for further reduction. Functionally, BCVA showed a different pattern. In within-group analyses, significant improvement from baseline to 4 months was observed only in pachychoroid eyes (*P* = 0.012), but the time-by-pachychoroid interaction was not significant (*P* = 0.315), indicating no between-group difference in visual improvement. Between-group BCVA did not differ significantly at baseline or at 4 months (*P* = 0.189 and *P* = 0.118). This apparent within-group difference is most likely attributable to a ceiling effect, as non-pachychoroid eyes had better baseline BCVA (0.18 vs. 0.37 logMAR), together with the limited subgroup size.

**Fig. 5:**
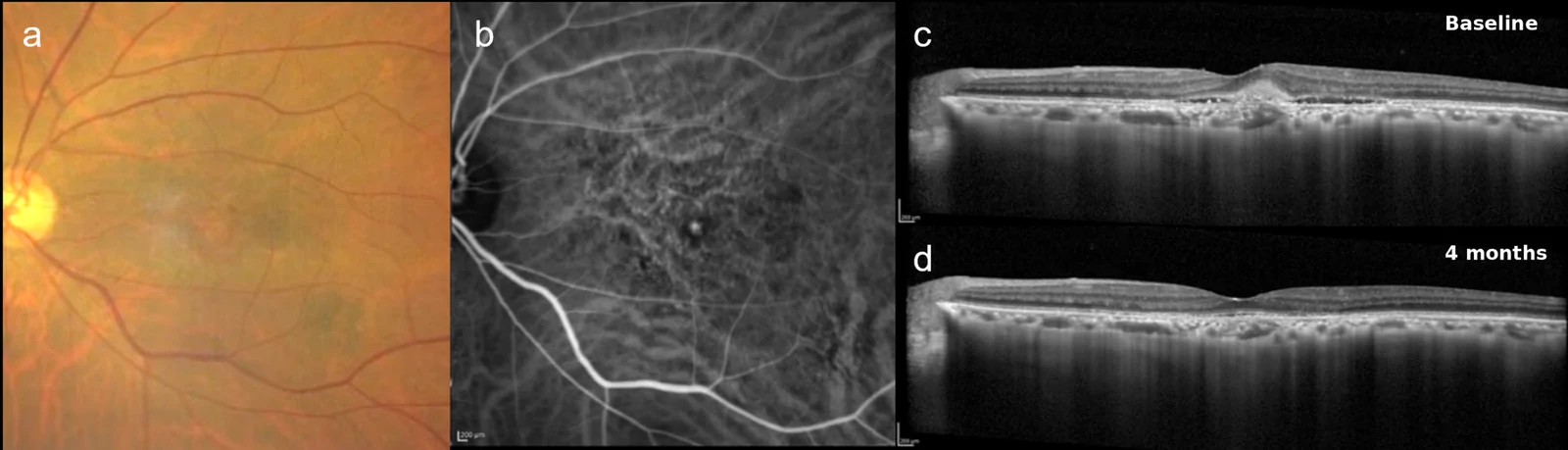
Representative multimodal imaging of an eye with polypoidal lesions showing resolution of subretinal hyperreflective material (SHRM) after three monthly loading injections of intravitreal aflibercept 8 mg. (**a**) Baseline color fundus photograph. (**b**) Baseline indocyanine green angiography (ICGA) showing a branching vascular network with a polypoidal lesion. (**c**) Baseline optical coherence tomography (OCT) B-scan through the fovea showing SHRM. (**d**) At 4 months, the SHRM had completely resolved. ICGA, indocyanine green angiography; OCT, optical coherence tomography; SHRM, subretinal hyperreflective material

For polypoidal lesion status, no significant differences in the time course of retinal or choroidal thickness were observed. However, for BCVA, the difference in time course by polypoidal lesion status was borderline (*P* = 0.052), and within-group analyses showed a significant BCVA improvement in eyes with polypoidal lesions but not in those without. At both baseline (Table 2) and 4 months (Table 3), there were no significant between-group differences in BCVA, retinal thickness, choroidal thickness, or the proportions of eyes with SRF or IRF. In contrast, at 4 months SHRM was absent in all 18 eyes with polypoidal lesions, including all 6 polypoidal eyes that had baseline SHRM, whereas SHRM was present in 6 of 27 eyes without polypoidal lesions (22.2%); this difference did not reach statistical significance (*P* = 0.067). Because the relevant time-by-phenotype interactions were not significant, a significant change within one subgroup but not in the other should not be interpreted as a true difference between subgroups; a genuine between-subgroup difference would require a significant interaction term. SHRM is a heterogeneous structure comprising fibrin, exudates, hemorrhage, neovascular tissue, and fibrosis; persistent foveal SHRM has been associated with poorer visual outcomes even after the resolution of intraretinal and subretinal fluid [28], and fibrotic SHRM frequently persists despite anti-VEGF therapy [29]. The absence of residual SHRM in all polypoidal-lesion eyes at 4 months was an exploratory observation, and prior reports using aflibercept 8 mg have documented high polyp regression rates (72.7–83%) [9, 10]. Moreover, meaningful structural remodeling or regression of SHRM often requires longer observation than the 4 months evaluated here. In addition, in PCV, the polypoidal lesions and branching vascular network are generally sub-RPE structures, whereas SHRM is an above-RPE OCT finding; therefore, the disappearance of SHRM should not be equated with regression of the underlying polypoidal lesion. The SHRM resolution we observed likely reflects, at least in part, resolution of its exudative and fibrinous components; whether regression of the underlying polypoidal lesion also contributed could not be determined, because post-treatment ICGA was not performed. Given this and the short 4-month follow-up, any relationship between SHRM resolution, visual improvement, and polyp regression remains speculative and hypothesis-generating. A representative case is shown in Fig. 5.

Although PCV is widely regarded as part of the pachychoroid disease spectrum [30, 31], only 5 of the 18 polypoidal-lesion eyes in our cohort (27.8%) met our pachychoroid criteria. In our cohort, the polypoidal-lesion eyes had, on average, a relatively thin choroid (mean baseline CCT 238.7 μm; median 190 μm), with 13 of 18 below 250 μm, so most fell below the 267.5 μm subfoveal choroidal-thickness cutoff reported to distinguish pachychoroid from non-pachychoroid PCV; they were also older (mean age 77.4 years) than the pachychoroid PCV subtype, which has a reported mean age of 64 years [32]. Consistent with this, the polypoidal-lesion eyes without pachychoroid features were older (mean 80.4 vs. 69.6 years) and had a thinner choroid (207.8 vs. 319.0 μm) than those with pachychoroid features; because choroidal thickness decreases with age, age-related choroidal thinning may attenuate pachychoroid features over time. Together, these findings indicate that most of our polypoidal-lesion eyes belonged to the non-pachychoroid (thinner-choroid, often older) PCV phenotype, consistent with the recognized heterogeneity of choroidal thickness in PCV.

Matsumoto et al. reported non-infectious intraocular inflammation associated with retinal vasculitis in 3 of 35 eyes (8.6%) at 4 weeks after the initial injection in their initial report [33]; the same group subsequently reported a similar incidence of 10.8% (9 of 83 eyes) during the loading phase in a larger consecutive cohort [9]. Cattaneo et al. [11] reported one intraocular inflammation case (10%) among 10 eyes in their Swiss cohort. In the PULSAR trial, the incidence of non-infectious intraocular inflammation was 1% in each arm [6]. In contrast, Hosoda et al. reported no cases of intraocular inflammation in a Japanese real-world cohort of 21 eyes [10], and we similarly did not observe non-infectious intraocular inflammation, retinal vasculitis, or other serious ocular adverse events in our 45 eyes during the 4-month follow-up. These discrepancies may be explained by differences in follow-up duration and sample size. Patients should be carefully monitored for intraocular inflammation, including retinal vasculitis on fundus examination, throughout treatment, particularly during the loading phase, when most reported cases have occurred. Systemic adverse events were not evaluated.

### Limitations

This study has several limitations. First, the retrospective design and the small sample size may limit generalizability. Second, the follow-up period was short, and long-term outcomes were not assessed. Third, the small sample size within each subgroup provided limited statistical power to detect time-by-phenotype interactions, and a type II error cannot be excluded; we therefore emphasize the width of the confidence intervals rather than statistical significance. Fourth, exudative findings (SRF, IRF, and SHRM) were assessed only qualitatively, and inter-reader agreement was not formally measured. Fifth, although the mixed-effects models accounted for repeated measures and Bonferroni correction was applied to pairwise comparisons within each outcome, no adjustment was made for the multiple outcomes (BCVA, CRT, CCT) and subgroup analyses examined, which may inflate the overall type I error rate. Sixth, although this was a multicenter study and sensitivity analyses confirmed the consistency of the principal findings, bias related to differences in patient populations between the two hospitals cannot be fully excluded. Seventh, baseline characteristics differed between subgroups, particularly choroidal thickness; although we performed an additional analysis adjusting for baseline CCT, other baseline differences and unmeasured factors that could affect treatment response could not be fully accounted for. Finally, post-treatment ICGA and OCTA were not performed, precluding direct assessment of polyp regression or of the angiographic correlates of SHRM resolution. In addition, quantitative CRT and CCT measurements were performed manually by a single masked retinal specialist, and intra- and inter-grader reproducibility of these measurements was not formally assessed.

## Conclusions

In conclusion, intravitreal aflibercept 8 mg led to significant short-term improvements in functional and anatomical outcomes in this real-world, multicenter Japanese cohort of treatment-naïve eyes with nAMD. The functional and anatomical benefit was broadly consistent irrespective of pachychoroid or polypoidal lesion status. The greater within-group visual improvement and absence of residual SHRM observed in eyes with polypoidal lesions are exploratory, hypothesis-generating observations that warrant confirmation in larger, longer-term studies.

## Data Availability

No datasets were generated or analysed during the current study.

## Declarations

### Use of generative AI and AI-assisted technologies in the writing process

During the preparation of this work, the authors used DeepL and Grammarly to assist with translation from Japanese to English and to perform grammar checking, respectively. After using these tools, the authors reviewed and edited the content as needed and took full responsibility for the content of the publication.

## Abbreviations

AMD: Age-related macular degeneration
BCVA: Best-corrected visual acuity
CCT: Central choroidal thickness
CRT: Central retinal thickness
EDI: Enhanced-depth imaging
EMM: Estimated marginal means
FA: Fluorescein angiography
ICGA: Indocyanine green angiography
IRF: Intraretinal fluid
logMAR: Logarithm of the minimum angle of resolution
MNV: Macular neovascularization
nAMD: Neovascular age-related macular degeneration
OCT: Optical coherence tomography
PCV: Polypoidal choroidal vasculopathy
PNV: Pachychoroid neovasculopathy
SD-OCT: Spectral-domain optical coherence tomography
SHRM: Subretinal hyperreflective material
SRF: Subretinal fluid
STROBE: Strengthening the Reporting of Observational Studies in Epidemiology
VEGF: Vascular endothelial growth factor
